# Vacuum and Infrared-Assisted Hot Air Impingement Drying for Improving the Processing Performance and Quality of *Poria cocos* (Schw.) Wolf Cubes

**DOI:** 10.3390/foods10050992

**Published:** 2021-05-01

**Authors:** Weipeng Zhang, Chang Chen, Zhongli Pan, Zhian Zheng

**Affiliations:** 1 School of Artificial Intelligence, Beijing Technology and Business University, 11 Fucheng Road, Beijing 100048, China; zhangwp@btbu.edu.cn; 2 Department of Biological and Agricultural Engineering, University of California, Davis, One Shields Avenue, Davis, CA 95616, USA; cgchen@ucdavis.edu (C.C.); zlpan@ucdavis.edu (Z.P.); 3 College of Engineering, China Agricultural University, 17 Qinghua Donglu, Beijing 100083, China

**Keywords:** vacuum, physicochemical properties, poria cubes, optimization, stage drying

## Abstract

The objective of this study was to develop an efficient drying technology for poria cubes in order to improve product quality. Poria cubes were dried using different methods, including air impingement drying, infrared-assisted air impingement drying, vacuum drying, two-stage vacuum drying, and infrared-assisted air impingement drying. The results were compared with those from hot air drying. For the two-stage drying, the tested conditions were the first stage of vacuum drying with temperatures between 65–85 °C and a switching moisture ratio of 70–90%. The second stage infrared-assisted air impingement drying also had temperatures 65–85 °C. The drying kinetics (effective moisture diffusivity (*D_eff_*), Biot number (*Bi*), and mass transfer coefficient (*k*) were studied via the product qualities (broken ratio, firmness, microstructure, and water-soluble polysaccharide content) and specific energy consumption (SEC) of the drying processes. The results showed that two-stage drying led to the lowest drying time and energy consumption, and also obtained the best qualities. Box–Behnken experimental design with response surface methodology (RSM) was used to optimize the two-stage operating conditions as 82 °C under vacuum drying until a moisture content of 81% and a temperature of 69 °C with infrared-assisted air impingement drying was achieved. These findings suggested that two-stage vacuum and infrared-assisted air impingement drying is a promising method for producing high quality and energy efficient dried poria cubes.

## 1. Introduction

*Poria cocos* (Schw.) Wolf is a fungus that is usually found on the roots of pine trees that grow in mountainous and hilly regions in the southern China. It is a traditional herb and is commonly used for treating insomnia, urinary dysfunction, and cancer [1]. Pharmacological studies have identified that the functionality and biomedical activities of *poria cocos* should be attributed to water-soluble polysaccharides, including (1,3)-β-D-glucan, (1,3)-α-D-glucan (1,3)-β-D-glucose, and more [2]. Nowadays, poria cubes or slices are consumed as an important daily functional food for its health benefit. 

Due to the high moisture content and perishability, freshly harvested *poria cocos* is vulnerable to quality deterioration and spoilage. Drying is an essential operation in the postharvest processing of *poria cocos* products because it prevents microorganisms to grow and extends shelf life. The quality attributes of poria cubes are usually affected during the drying process. Particularly, dried poria cubes that are broken or cracked usually have particles diameter of less than 5 mm and much lower market prices than intact ones [3]. In addition, dried products with low contents of water-soluble polysaccharides have fewer nutritional values, which are less acceptable to consumers.

Hot air drying (HAD) is one of the most commonly used drying methods for poria cubes due to its low equipment cost and simplicity. However, it causes the non-uniform distribution of heat and moisture in materials, thus resulting in the breaking and cracking of poria cubes. The HAD process is also time-consuming and energy-intensive, which leads to low processing throughputs and high production costs [4,5]. The demands for higher drying efficiency, lower energy consumption, and better product quality have led to increasing interests in developing alternative drying methods for poria cubes.

Compared to the HAD, the air impingement drying (AID) process is worth examining. Therein, hot air impinges on the surface of materials at a high velocity, enhancing heat and mass transfer rates and rapidly removing the water from the material’s surface [6]. Deng et al. [7] observed that hot air impingement drying (AID) significantly reduced the thickness of the thermal boundary layer on the surface of pepper and shortened its drying time. Xiao et al. [8] considered AID as one of the fastest drying methods for materials with small dimensions. Moreover, the reduction in drying time was beneficial for saving energy during the drying process.

For infrared (IR) drying, thermal energy is transferred from the heating element to a product’s surface via radiation. Consequently, IR drying has the advantages of uniform heating, a low processing time, and a high heat transfer rate [9]. However, because of the limited penetration depth, IR may not be used as a single heating source during drying processes [10]. IR-assisted hot air impingement drying (IR-AID) adopts the advantages of both IR and AID, resulting as a uniform, rapid, and energy-efficient drying process [11]. Zhang et al. [12] reported that IR-AID was a promising drying technology for high-quality sponge gourd slices, as it significantly reduced its energy consumption. Supmoon [13] showed that IR-AID led to a higher drying rate, less shrinkage, lower hardness, and less color deterioration for the production of healthy potato chips. However, the study of IR-AID for poria cubes is still limited [3].

The presence of oxygen in the atmosphere during air drying usually has negative effects on the quality attributes of poria cubes, particularly causing the oxidation of water-soluble polysaccharides [14]. Therefore, vacuum drying (VD) may preserve the quality of poria cubes due to its oxygen-deficient drying conditions. However, the VD process is slow and consumes large amounts of energy, which may not be economically feasible for large-scale productions. Therefore, applying a two-stage drying method (i.e., VIR-AID) by using the VD and IR-AID may fuse the advantages of both drying methods, creating a synergistic effect for efficiently drying poria cubes with improved quality. The VIR-AID drying strategy method was proposed as a promising efficient drying method for poria cubes. Response surface methodology (RSM) is a widely used approach for optimizing [15] the drying processes of different types of foods, such as mushrooms [16], bee pollen [17], and potato slices [18]. Currently, there is no available information in the literature about the optimization of VIR-AID drying conditions for poria cubes.

The knowledge of drying kinetics is important for determining suitable operating conditions for improved drying efficiency [19]. Dincer and Hussain [20] developed a semi-analytical drying model with new drying parameters, namely drying coefficients and lag factors, which considered both external and internal resistances to moisture transfer. Mass transfer parameters—such as Biot (*Bi*) number, effective moisture diffusivity (*D_eff_*), and moisture transfer coefficient (*k*)—during the drying process could be determined in a simple and accurate manner. The drying model has been successfully applied to the drying kinetics of yam slices [21], apple slices [22], and passionfruit peel [23].

Therefore, the firmness, integrity, and water-soluble polysaccharide contents of dried poria cubes are used as key quality indicators in the poria processing industry. The objectives of this study were to: (1) study the drying characteristics, drying kinetics, product qualities, and energy consumption of poria cubes under HAD, AID, IR-AID, VD, and VIR-AID; and to (2) optimize the operating conditions of the VIR-AID method in order to minimize the total drying time, broken ratio, and SEC, as well as to maximize the water-soluble polysaccharide content via RSM.

## 2. Materials and Methods

### 2.1. Raw Material

**Figure 1:**
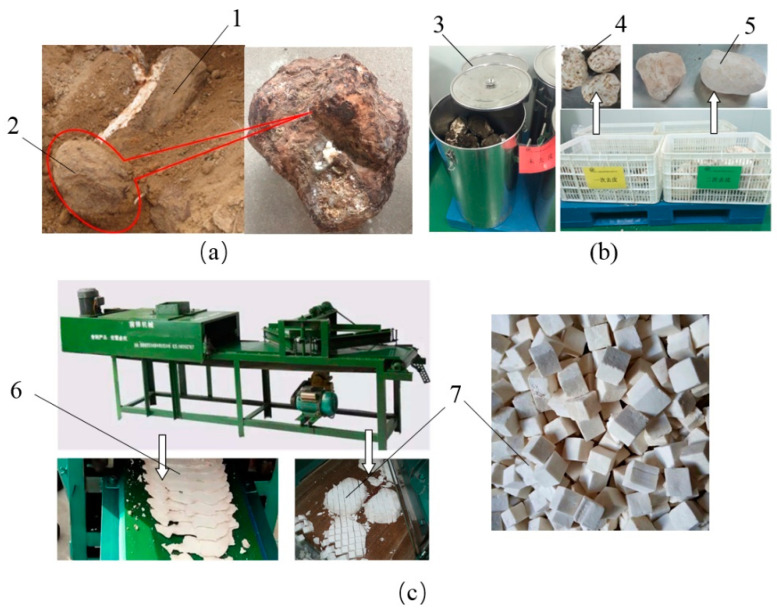
Production procedures of fresh poria cubes: (**a**) Raw material; (**b**) peeling process; (**c**) slicing and cubing processes. (1) Pine tree; (2) *poria cocos*; (3) stainless-steel container; (4) *poria cocos* after first peeling; (5) *poria cocos* chunks after second peeling; (6) poria slices; (7) poria cubes.

Freshly harvested *poria cocos* produced in Jinzai (Anhui, China) were provided by Qiaokang Technology Co., Ltd. Ripe *poria cocos* were manually dug out of the soil. The *poria cocos* with a uniform shape and weight of 2.9 ± 0.3 kg were selected for performing the experiments and stored in a refrigerator at 4 °C to maintain freshness and ensure consistency. Poria cubes were produced with a procedure shown in Figure 1. Specifically, whole fresh *poria cocos* were stored in a stainless-steel tub at room temperature for 2 h to reach a uniform initial temperature of 24 ± 2 °C. Then, the brown peel of *poria cocos* at the outer layer was manually removed with a knife during the first and second peeling processes. The white *poria cocos* chunks were obtained and covered with a polyethylene plastic film to avoid moisture loss. Poria cubes with the side length of 14 mm were produced using a cookie-cutter (Yuexi Mechanical Technology Co. Ltd., Jinzai, Anhui, China). Finally, the poria cubes without damaged edges and corners were selected for the drying experiments. The initial moisture content (*MC*_0_) of the poria cubes was 0.51 ± 0.01 kg·kg^−1^ on a wet basis (1.04 kg·kg^−1^ on dry basis), which was determined with a vacuum oven drying at 75 °C for 24 h according to the Official Analytical Chemists method no.934.06. Triplicate measurements were conducted.

### 2.2. Drying Equipment

**Figure 2:**
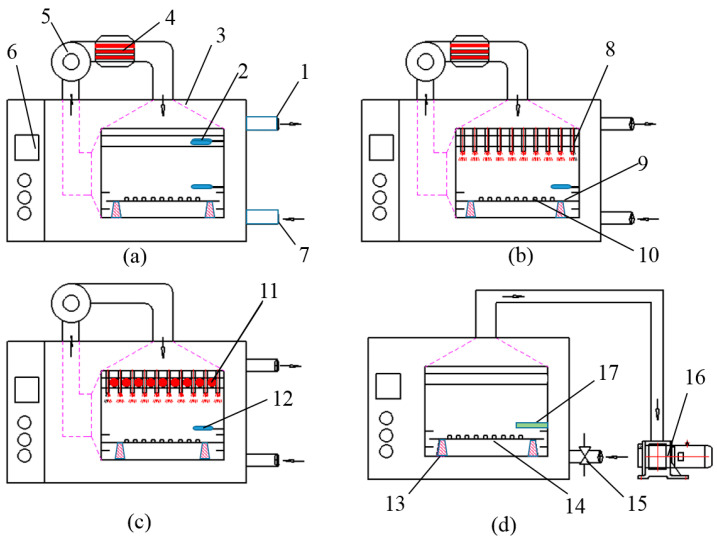
Schematic diagrams of lab-scale drying system: (**a**) HA dryer; (**b**) AID dryer; (**c**) IR-AID; and (**d**) VD dryer. (1) Air outlet; (2) temperature and humidity sensor; (3) air distribution chamber; (4) electric assistance; (5) centrifugal fan; (6) control system; (7) air inlet; (8) nozzles; (9) trays; (10) poria cubes; (11) IR emitters; (12) drying temperature sensor; (13) load cell; (14) electric heating board; (15) fast solenoid valve; (16) vacuum pump; (17) pressure sensor.

Drying tests were carried out using four high-precision computer-controlled drying systems at the processing workshop of Qiaokang Technology Co., Ltd. processing workshop (Anhui, China). As shown in Figure 2, digital load cell systems with a precision of 0.01 g (HYLF-010, Meikong, Hangzhou, China) were installed in the four dryers to track the weight change and moisture loss of samples during the drying process. The energy consumptions of different drying processes were measured by a watt-hour meter (DTSU1717-4P, HangLong, Shanghai, China). The change of weight and energy consumption were monitored and recorded by the logging system automatically and continuously.

The HA dryer shown in Figure 2a was mainly comprised of an electric heater and an air conditioning unit. An electric heater was installed between the fan and drying chamber to heat the air to the target temperature. A centrifugal fan was installed above the dryer to draw air from the air inlet and blow hot air into the drying chamber via an air distribution chamber. The airflow was measured using an anemometer (SW-6086, Suwei, Hangzhou, China) and adjusted by an inverter (EV4300, Taida, Shanghai, China). A thermocouple with an accuracy of ±0.1 °C was installed on the sample tray to measure and control the air temperature. As shown in Figure 2b,c, the air impingement system in the AID dryer and IR-AID dryer had similar configurations. Compared with the HA dryer, a series of round nozzles were installed in the air distribution chamber of the AID and IR-AID dryer. The centrifugal fan delivered the vertical airflow with 0.6 m·s^−1^ velocity, which was speed up to 3.0 m·s^−1^ as it passed through the nozzle and impinged the poria cubes. The distance between the nozzles and drying samples was fixed at 120 mm. Since the vertical airflow affected the load cell system’s measuring precision, the centrifugal fan stopped for 20 s when the sample was weighed every 15 min. For the IR-AID dryer, a row of infrared emitters (IR-0H03, Hongyao, Jiangshu, China) was installed on top of the tray to ensure uniform heating.

As shown in Figure 2d, the VD system mainly consisted of a drying chamber and a vacuum pump (2BV2070, Bosan, Shandong, China). The drying temperature of the electric heating board was controlled by a PID controller (model E5CN, Omron, Tokyo, Japan) with a sensitivity of ±0.5 °C. Chamber pressure was maintained at 5 ± 3 kPa and measured with a pressure sensor (MIK-P300, Meikong, Hangzhou, China). It had an accuracy of ±2 kPa. The boiling point of water under 5 kPa was nearly 33 °C [16].

### 2.3. Drying Experiments

The pre-drying temperature in all four drying scenarios was 65 °C. Before beginning the drying experiments, four dryers ran for 30 min to achieve a stable temperature. In each drying test, 5000 ± 5 g poria cubes were spread in a single layer on a stainless-steel tray with a loading capacity of 5 kg·m^−2^. The samples were dried to a final moisture content of 0.17 ± 0.005 kg·kg^−1^ on a dry basis for safe storage [24].

All experiments were repeated three times. After drying was finished, the dried poria cubes were cooled in a desiccator for 30 min. Then, they were vacuum-sealed in polyethylene bags to prevent moisture absorption. Further, they were stored in a refrigerator (4 °C) for no longer than 3 days.

### 2.4. Drying Kinetics

The moisture content (MCs) of poria cubes on a dry basis was determined in a vacuum oven at 75 °C for 24 h according to the official analytical chemists method No. 934.06. The moisture ratio (MR) and drying rate (DR) were calculated using the following equations [7]:

(1) $$\mathrm{MR}=\frac{MC_{t}-MC_{e}}{MC_{0}-MC_{e}}$$

(2) $$\mathrm{DR}=\frac{MC_{t_{1}}-MC_{t_{2}}}{t_{1}-t_{2}}$$

where *MC_t_* is the moisture content on a dry basis at a particular drying time *t*, kg·kg^−1^ dry mass. *MC*_0_ is the initial *MC* on dry basis, kg·kg^−1^ dry mass. *MC_e_* is the equilibrium *MC* under different drying methods, around 0.08 kg·kg^−1^ dry mass. *MC_t_*_1_ and *MC_t_*_2_ are the *MC*s of *poria cocos* cubes on a dry basis at drying times *t*_1_ and *t*_2_, respectively, kg·kg^−1^ dry mass. *t*_1_ and *t*_2_ are the minimum drying times.

Fick’s second law is widely used to describe the effective moisture diffusivity (*D_eff_*) with which one can make assumptions about neglected shrinkage, constant temperature, and diffusion coefficients, as well as uniform initial moisture distribution. *D_eff_* can be calculated using the following equations [8]:

(3) $$\mathrm{MR}=\frac{8}{\pi^{2}}\sum_{n=0}^{\infty }\frac{1}{{\left(2n+1\right)}^{2}}\exp\left[-\frac{{\left(2n+1\right)}^{2}\pi^{2}D_{eff}}{4L^{2}}t\right]\approx \frac{8}{\pi^{2}}\exp(-\frac{\pi^{2}D_{eff}}{4L^{2}}t)$$

(4) $$K_{0}=-\frac{\pi^{2}D_{eff}}{4L^{2}}$$

where *D_eff_* is the effective moisture diffusivity, m^2^·s^−1^. *n* is an integer. *L* is the sample thickness, m. *t* is the drying time, s. $K_{0}$ is calculated by plotting ln (MR) versus time *t*.

### 2.5. Dincer Drying Model

The drying kinetics of poria cubes under different drying methods were studied using the Dincer model. Specifically, the experimental data of *MR*s were inserted into Equation (3).

According to the Dincer model, *MR* could be expressed in the exponential equation as follows [20]:

*MR* = *G*·exp(−*St*) (5)

where *G* is the lag factor, indicating the resistance of moisture transfer during drying, and *S* is the drying coefficient, 1·s^−1^, indicating the drying capability of wet materials.

The effective moisture diffusivity (*D_eff_*, m^2^·s^−1^) was calculated based on the developed relationship [21]:

*D_eff_* = *S*(*L*/*μ*_1_)^2^ (6)

The coefficient *μ*_1_ was determined by the following equation [25]:

*μ*_1_ = −419.24*G*^4^ + 2013.8*G*^3^ − 3615.8*G*^2^ + 2880.3*G −* 858.94 (7)

The mass transfer Biot number was determined using the *Bi*-*G* correlation [26]:

*Bi* = 0.0576*G*^26.7^ (8)

The moisture transfer coefficient (*k*, m·s^−1^) was determined as follows [27]:

*k* = *D*·*Bi*/*L* (9)

The goodness of fir for the Dincer model was evaluated based on the adjusted coefficient of determination (*adj*-*R*^2^), reduced chi-square (*χ*^2^), and the root mean square error (RMSE) [28]. The qualified fit should have the highest *adj*-*R*^2^ and lowest *χ*^2^. The nonlinear curve that fit to the experimental data was performed using the MATLAB software (Math Works Inc., Model-R2013a, Natick, MA, USA).

(10) $$adj\text{-}R^{2}=1-\frac{\left(1-\frac{\sum {}_{i=1}^{N}{\left(MR_{pre,i}-MR_{exp,i}\right)}^{2}}{\sum {}_{i=1}^{N}{\left(MR_{pre,i}-\bar{MR}\right)}^{2}})(N-1\right)}{N-k-1}$$

(11) $$\chi^{2}=\frac{\sum {}_{i=1}^{N}{\left(MR_{exp,i}-MR_{pre,i}\right)}^{2}}{N-n}$$

(12) $$RMSE={\left[\frac{\sum {}_{i=1}^{N}{\left(MR_{exp,i}-MR_{pre,i}\right)}^{2}}{N}\right]}^{1/2}$$

where $M_{pre,i}$ is the *i*th predicted *MR*, $M_{exp,i}$ is the *i*th experimental *MR*, $\bar{MR}$ is the mean of the experimental *MR*, and *N* is number of observations. *N* and *k* are the number of constants and independent variables in the drying model, respectively.

### 2.6. Response Surface Methodology

Based on the drying experiments, a two-stage drying strategy using VD followed by IR-AID was proposed as a promising and efficient drying method for poria cubes. In the first stage, VD was applied to dry the poria cubes to a critical moisture ratio (*MR*) before the cubic shape started to significantly change. Then, the IR-AID method was used to complete the drying in the second stage.

A three-level Box–Behnken experimental design with three factors was applied to study the two-stage VIR-AID process of poria cubes. The heating temperature during the VD stage, the sample moisture ratio (*MR*) at the time of switching the drying method, and the heating temperature during the IR-AID stage were selected as our independent experimental variables. The factor levels were selected as follows: VD temperature (*T*_VD_, 65–85 °C), *MR* at the switch point (*MR*_switch,_ 70–90%), and IR-AID temperature (*T*_IR-AID_, 65–85 °C). The response variables were the overall drying time (VD time + IR-AID time), broken ratio, water-soluble polysaccharide content, and SEC of poria cubes. The experimental design consisted of 17 experiments with 5 replicates at the central point. As shown in Table 1, the independent variables were coded with values of −1, 0, and 1.

The second order polynomial coefficients were calculated and analyzed using the Design Expert software v.10 Trial (Stat-Ease, Minneapolis, MN, USA). The general form of the second-degree polynomial was as follows [5]:

(13) $$Y=\beta_{0}+\sum {}_{i=1}^{n}\beta_{i}x_{i}+\sum {}_{i=1}^{n}\beta_{ii}x_{i}^{2}+\sum {}_{i=1}^{n-1}\sum {}_{j=i+1}^{n}\beta_{ij}x_{i}x_{j}$$

where *Y* is the predicted response, *x_i_* and *x_j_* are input variables input variables that influence the response variable *Y*; *β*_0_ is the offset term; *β_i_*, *β_ii_*, and *β_ij_* are the linear coefficient, quadratic coefficient, and interaction coefficient, respectively.

The numerical optimization of process variables based on multiple responses was performed using the Design Expert software. Desired goals (minimization of drying time, broken ratio, SEC, and the maximization of water-soluble polysaccharide content) were used to perform optimization of factors and the response.

### 2.7. Broken Ratio

**Figure 3:**
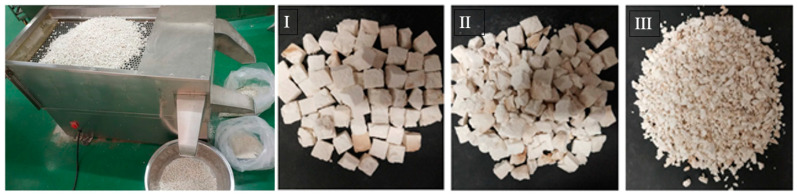
Vibrating sieve and typical images of I, II, and III grade products.

The dried poria cubes were sorted by using a vibrating sieve (MGSXJ-12W, Guanghe, China), as shown in Figure 3. Based on the dimensions of samples, the dried poria cubes were classified into three grades: I, II, and III. The shape of grade I products was nearly cubic with slight side and corner damage. For grade II, obvious breakage and cracking were observed. Broken particles with diameters less than 5 mm were defined as grade III products.

The broken ratio (*μ*,%) was described using the following equation:

(14) $$\mu =\frac{m_{\mathrm{II}}+m_{\mathrm{III}}}{m_{I}+m_{\mathrm{II}}+m_{\mathrm{III}}}$$

where *m*_I,_ *m*_II,_ and *m*_III_ were the sample masses (kg) of I, II, and III grade poria cubes, respectively.

### 2.8. Firmness

The firmness of poria cubes was determined via the compression test using a texture analyzer (TAPlus, Godalming, Surrey, UK). After sorting, the grade I poria cubes were randomly selected for the compression test. The diameter of two cylindrical probes was 35 mm and the compression parameters were as follows: maximum load 500 N, compression distance 8 mm, trigger force 0.1 g, pre-test speed 5 mm·s^−1^, test speed 1 mm·s^−1^, and post-test speed 5 mm·s^−1^. The force-distance curve was recorded and the load force of the first peak was defined as the firmness. The compression tests for each drying condition were repeated 30 times and the average value was calculated and reported.

### 2.9. Water-Soluble Polysaccharide Content

The water-soluble polysaccharide content was determined via the phenol-sulfuric acid method by using D-glucose as the standard [29]. Specifically, dried poria cubes were ground up and passed through a 60-mesh screen. The powder (0.5 ± 0.001 g) was mixed with 25 mL distilled water in a 50 mL volumetric flask. The suspension was then heated in a 60 °C water bath with ultrasonic for 30 min to accelerate the extraction rate of polysaccharides, and then cooled to room temperature. The solution was vacuum filtrated to remove the solid residual. The solid residual was then thrice washed with distilled water. The washed water was then combined with the filtrated solution and diluted to 100 mL (*v*_1_). Next, 0.5 mL(*v*_2_) of filtrated solution was pipetted into a 10 mL tube and 1 mL of 5% phenol was added. Then, the mixture was shaken for 2 min. Further, 5 mL of sulfuric acid (98% *v*·*v*^−1^) was added into the mixture and shaken for 5 min. The absorbance at 490 nm was then determined using a UV-Vis spectrophotometer (Shimadzu UV-2600, Hangzhou, Zhejiang, China). Distilled water was used as a blank. The total water-soluble polysaccharides content (*φ*, mg·g^−1^) of the dried poria cubes was calculated with the following equation:

(15) $$\phi =\frac{200\cdot f}{m_{1}}\cdot (\frac{y-0.0882}{7.5486})$$

where *y* is the absorbance of the filtrated solution at 490 nm; m_1_ is the mass of the poria powder sample, 5 g; and *f* is the conversion factor, 1.28 [14].

### 2.10. Specific Energy Consumption

The specific energy consumption (SEC, MJ·kg^−1^), which was the energy needed to remove 1 kg of water from poria cubes was calculated using Equation (16) [28]:

(16) $$SEC=\frac{E}{m_{water}}$$

where *E* is the energy consumed during drying, MJ, and *m_water_* is the mass of moisture removal during the drying, kg.

### 2.11. Statistical Analysis

The experimental data were calculated as means ± standard deviation (SD). Analysis of variance (ANOVA) was performed using Duncan’s multiple range test with a significance level of 0.05. The *F* test was carried out to assess the homoscedasticity of the residuals by using the Design Expert software v.10 trial (Stat-Ease).

## 3. Results and Discussion

### 3.1. Drying Characteristics of Poria Cubes under Different Drying Methods

**Figure 4:**
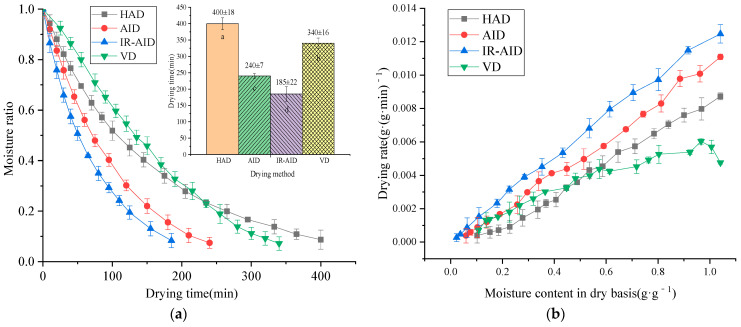
*MR* curves and drying rate curves of poria cubes under different drying methods: (**a**) *MR* curves; (**b**) drying rate curves.

As shown in Figure 4a, the *MR* of poria cubes exponentially decreased with drying time, which was commonly observed during the food drying process [30]. The times required to dry the poria cubes from their initial *MC*s (1.04 kg·kg^−1^ on dry basis) to the desired final *MC*s (0.17 kg·kg^−1^ on dry basis) for HAD, AID, IR-AID, and VD were 400 min, 240 min, 185 min, and 340 min, respectively. Compared with the HAD, the AID and IR-AID reduced the drying time by 32.5% and 46.3%, respectively. Such results should mainly be attributed to air impingement, which sped up the airflow and significantly decreased the thicknesses of the heat and mass transfer boundary layer between the material surface and air, thus improving the drying rate. The results showed similar trends as reported in previous studies on the drying of Monukka seedless grapes [31]. In addition, the drying time when using IR-AID was 55 min shorter than when using AID, which could be attributed to the improved heating intensity and rate of moisture evaporation due to IR heating.

The drying rate of VD was lower than HA drying during the early stage. It then became higher later. The VD drying time was shorter than the HA drying time. Such results were mainly due to the shape of the poria cubes, which remained intact during VD due to the lower temperature gradient and less thermal strain. While under HA, AID, and IR-AID, the poria cubes showed apparent cracking and braking, which contributed to more exposed surfaces for moisture evaporation when drying began. Similar findings were observed by Seremet et al. [32], who found that pumpkin slice structure changed under different drying methods, significantly affecting drying time. As shown in Figure 4b, drying occurred mainly in the falling-rate stage, except for the VD, where a short period of acceleration was observed when drying began. The moisture inside the poria cubes could be classified into intercellular water (loosely bonded water) and intracellular water (strongly bound water) [33]. The higher drying rates during the initial drying stages could be due to the abundant intercellular water in the poria cubes. During later drying stages, the drying rates were mainly controlled by the diffusion of intracellular water, which had a higher resistance to be removed when dried. Similar trends were observed in the HAD of apple and potato cubes, according to Khan et al. [34].

### 3.2. Drying Kinetics

The Dincer model (Equation (5)) was used to fit the drying curves under different drying methods. The lag factors (*G*) and drying coefficients (*S*) were determined and summarized (Table 2). The high values of *adj-R*^2^ and low values of *χ*^2^ and RMSE indicated good fits of the model. The lag factor (*G*) was directly related to the mass transfer Biot number (*Bi*), as expressed in Equation (8). *Bi* is one of the most important dimensionless numbers in drying that represents the magnitude of resistance to moisture diffusion inside the material. *Bi* < 0.1 indicates negligible internal resistance, whereas *Bi* > 100 indicates negligible external resistance. In general, *Bi* numbers were ranged between 0.1 < *Bi* < 100 [20]. In this study, the calculated *Bi* values ranged between 0.1030 to 0.5626, indicating that both internal and external resistance to the moisture transfer existed during the poria cubes drying processes [22]. The drying coefficients (*S*) varied from 1.036 × 10^−4^ to 2.197 × 10^−4^ (1·s^−1^), which represented the drying capability of poria cubes. The infrared heating and hot air impingement drying enhanced drying capability, as indicated by the higher *S* values in AID and IR-AID.

*D_eff_* characterizes the rate of moisture diffusion within the food material [35]. As shown in Table 1, the magnitudes of *D_eff_* during the poria cube drying processes under HAD, AID, IR-AID, and VD were 8.148 × 10^−9^, 5.768 × 10^−8^, 7.901 × 10^−9^, and 8.401 × 10^−9^ m^2^·s^−1^, respectively. Meanwhile, the mass transfer coefficient (*k*) ranged from 6.385 × 10^−7^ to 1.162 × 10^−6^ m·s^−1^. Among the four drying methods, the highest effective moisture diffusivity was obtained from the IR-AID process, which showed the best potential to improve the drying efficiency of poria cubes. The *D_eff_* calculated by Fick’s second law ranged from 8.335 × 10^−9^~3.767 × 10^−8^ m^2^·s^−1^, which had a similar trend compare with the results of the Dincer drying model. The *D_eff_* was in the order of IR-AID > AID > VD > HAD. The difference of *D_eff_* determined from the Dincer and Fickian models may have been caused by the different mathematical models. This phenomenon was consistent with the drying of kiwifruit slices. Moreover, the specific values of *D_eff_* calculated via the Dincer drying model and Fick’s second law are not the same [25].

### 3.3. Broken Ratio and Firmness

**Figure 5:**
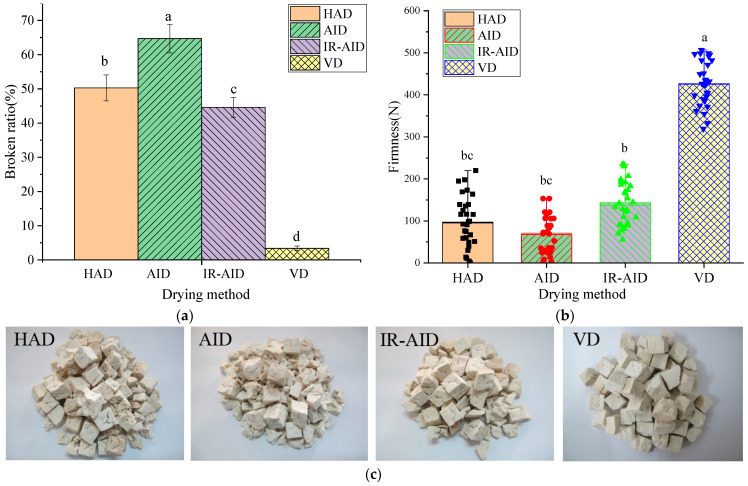
Broken ration, firmness, and images of poria cubes under different drying methods: (**a**) Broken ratio; (**b**) firmness; (**c**) images of poria cubes under different drying method. Different lowercase superscripts denote significant differences (*p* < 0.05).

The broken ratio was used to characterize the degree of structural destruction of dried poria cubes. Usually, the firmness of a material had a major influence on the broken ratio, and dried products with higher firmness usually had a lower broken ratio [36]. Figure 5a,b shows the broken ratio and firmness of poria cubes obtained from the four drying methods. The average broken ratios of dried poria cubes were 50.3%, 64.7%, 44.6%, and 3.4% under HAD, AID, IR-AID, and VD, respectively. The average firmness values were 9.94, 6.91, 14.65, and 43.48 kg for HA, AID, IR-AID, and VD, respectively. Meanwhile, Figure 5c shows the appearance of the dried poria cubes as a result of different drying methods. The results showed that poria cubes dried via the AID method had the highest broken ratio and lowest firmness. That was due to rapid airflow, which caused the material’s surface to quickly dry and stress contraction on the sample surface to intensify in the early stage of drying. The non-uniform distribution of temperature and moisture in poria cubes led to severe surface cracking. The broken ratio of IR-AID was nearly 20% lower than that of the AID samples. Such results should be attributed to the improvement of heating uniformity within poria cubes via IR heating [37]. The gradients of temperature and moisture within poria cubes were reduced and thus cracking was mitigated. The broken ratio of HAD dried samples was nearly 10% lower than that from the AID, which was mainly due to the lower drying rates and less moisture gradient within the poria cubes. The lowest broken ratio and highest firmness were obtained in VD samples. As shown in Figure 5c, the VD samples had intact cube shapes without obvious damaged edges or corners. The results should mainly be attributed to the fact that the material temperature was typically low, as vacuums tend to reduce the boiling point of water or solvents, thus reducing thermal stresses and over-drying caused by high material temperature [38]. This improved the firmness and texture of dried poria cubes. Compared with the other three drying methods, the lower drying rate during VD also decreased the moisture gradient and the internal stress concentration in poria cubes. Due to the significant reduction of the boiling point of water, VD has been widely applied to eliminate internal fissures and bubbles in drying samples [39].

The better integrity and higher firmness of VD-dried poria cubes should also be attributed to the uniform volume shrinkage during the drying process [40]. During the food drying process, volumetric shrinkages mainly occurred in two stages: major shrinkage occurred during the preheating and constant-rate drying period, and minor shrinkage subsequently occurred during the falling-rate drying period [41]. During the initial drying stage of VD, the moisture rapidly evaporated and the volume shrinkage of the poria cubes was proportional to the volume of moisture evaporation [42]. The end of the first stage was marked by a critical *MR* in the poria cubes. Below the critical *MR*, the volumetric change of poria cubes was minor and no longer proportional to the volume of moisture evaporation, and the cracking and breaking were also minor. At this stage, the VD should be switched to other rapid drying methods to reduce the overall drying time [43].

### 3.4. Retention of Water-Soluble Polysaccharide Contents

**Figure 6:**
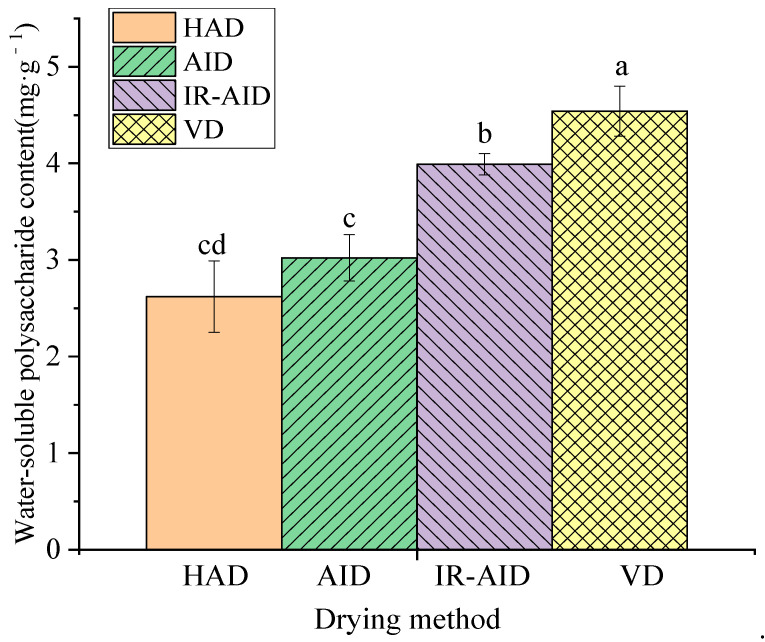
Water-soluble polysaccharide content of different drying methods. Different lowercase superscripts denote significant differences (*p* < 0.05)

Figure 6 shows the contents of water-soluble polysaccharide in the dried poria cubes under different drying methods. HAD resulted in the lowest water-soluble polysaccharide content of 2.62 mg·g^−1^. The water-soluble polysaccharide contents in the AID and IR-AID dried samples were 13.26% and 52% higher than that of HAD. Such results could be due to the prolonged exposure to oxygen during the air drying (HAD, AID, and IR-AID), causing the oxidative degradation of water-soluble polysaccharides [44]. During VD, the exposure of poria cubes to oxygen was limited, which was helpful for the retention of water-soluble polysaccharides and preservation of bioactive activities. Such results showed similar trends as reported by Yan et al. [45], who found that vacuum drying benefited the retention of water-soluble polysaccharides in bitter gourd (*Momordica charantia* L.) slices. Liu et al. [46] also proved that the physicochemical and biological activities of polysaccharides in Lentinula were strongly dependent on the type of drying method deployed.

### 3.5. Specific Energy Consumption

**Figure 7:**
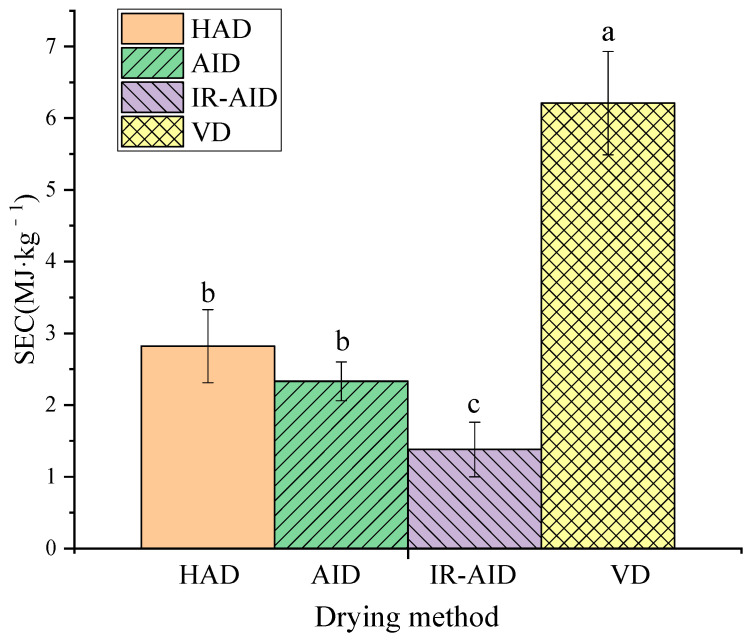
Specific energy consumption of different drying methods. Different lowercase superscripts denote significant differences (*p* < 0.05).

As shown in Figure 7, the SEC of the HAD, AID, IR-AID, and VD processes were 2.82, 2.33, 1.38, and 6.21 MJ·kg^−1^, respectively. The VD process resulted in the highest SEC, which was likely due to the longer drying time and high energy consumption of the vacuum pump. It is worth noting that IR-AID consumed the lowest energy, which was directly associated with its shortest drying time. Besides, IR radiation could be directly absorbed by samples with minimum ambient, which usually leads to higher energy efficiency [47]. Chen et al. [48] reported that infrared heating could be used in conjunction with HA drying to improve drying efficiency and reduce energy consumption of carrot snacks. VD resulted in the highest retention of water-soluble polysaccharides and the best integrity in poria cubes. IR-AID led to the lowest energy consumption and drying time. Therefore, a two-stage drying strategy using VD and followed by IR-AID was proposed for poria cubes.

### 3.6. Two-Stage Vacuum and Infrared-Assisted Hot Air Impingement Drying

Based on the results of the single factor experiments, a three-level Box–Behnken experimental design with three factors was applied to study the two-stage VD and IR-AID processes of poria cubes. As shown in Table 3, the total drying time, broken ratio, water-soluble polysaccharide content, and SEC ranged from 172–343 min, 3.79–44.20%, 1.25–4.45 mg·g^−1^, and 1.24–4.04 MJ·kg^−1^, respectively.

The response variables were fitted with second-order polynomial models using the least square method (MLS). The estimated regression coefficients, adjusted coefficient of determination (*Adj*-*R*^2^), and coefficient of variation (*C.V.*) values are summarized in Table 4. The *Adj*-*R*^2^ values on total drying time, broken ratio, water-soluble polysaccharide content, and SEC were all higher than 0.97, indicating that the polynomial models fit well with the experimental results. Moreover, the lack of fit test results showed that there was no lack of fit for the models. Relatively low values (<10%) of C.V. for all response variables implied a high degree of precision and good reliability of the experimental values.

The regression equations between response variables and coded factors were also established. The total drying time (*Y*_1_, min), broken ratio (*Y*_2_, %), water-soluble polysaccharide content (*Y*_3_, mg·g^−1^), and SEC (*Y*_4_, MJ·kg^−1^) are shown in Equations (17)–(20).

(17) $$Y_{1}=246.2-17.63x_{1}-55.37x_{2}-25.50x_{3}+2.75x_{1}x_{2}+1.00x_{1}x_{3}+1.52x_{1}^{2}+5.52x_{2}^{2}+5.78x_{3}^{2}$$

(18) $$Y_{2}=21.47-0.24x_{1}+19.76x_{2}+0.26x_{3}-0.01x_{1}x_{2}+0.03x_{1}x_{3}-0.16x_{2}x_{3}-0.49x_{1}^{2}+2.96x_{2}^{2}-0.079x_{3}^{2}$$

(19) $$Y_{3}=3.01+0.017x_{1}-0.052x_{2}-0.956x_{3}+0.17x_{1}x_{2}+0.41x_{1}x_{3}-0.21x_{2}x_{3}-0.27x_{1}^{2}+0.48x_{2}^{2}-0.2x_{3}^{2}$$

(20) $$Y_{4}=8.20-1.31x_{1}-2.94x_{2}-0.68x_{3}+0.9x_{1}x_{2}-0.036x_{1}x_{3}+0.045x_{2}x_{3}+0.36x_{1}^{2}+1.20x_{2}^{2}-1.09x_{3}^{2}$$

where $x_{1}$ represents *T*_VD_, °C, $x_{2}$ represents *MR*_switch_, %, and $x_{3}$ represented *T*_IR-AID_, °C.

The regression analysis results of Equation (17) showed that drying time was significantly affected by linear terms of *T*_VD_, *MR*_switch_, and *T*_IR-AID_. An increase of VD temperature and IR-AID drying temperature, as well as a decrease of *MR*_switch_, reduced the total drying time. The higher VD temperature and IR power could accelerate the water molecules present in the poria cubes, thus leading to faster evaporation and drying [12,16]. ANOVA results of Equation (18) suggested that *MR*_switch_ had a significant effect on the broken ratio of poria cubes during the VIR-AID drying process. The broken ratio of poria cubes decreased when *MR*_switch_ decreased. Table 3 also shows that when the *MR*_switch_ was higher than 80%, the broken ratio of products was higher than 20%. However, when *MR*_switch_ was 70%, the broken ratio of poria cubes was less than 5%. Such results should be due to the glass transition of poria cubes, from a rubbery state to a glassy state during the VD stage at an MR ranging between 70–80% [3]. Equation (19) showed that a lower VD temperature and *MR*_switch_ led to higher water-soluble polysaccharide content. This was consistent with the VD drying characteristics of poria cubes. Equation (20) indicated that SEC increased when *T*_IR-AID_ increased and decreased when *T*_VD_ and *MR*_switch_. In addition, the interaction terms between *T*_VD_ and *MR*_switch_ had a negative effect on SEC. Since the vacuum pump had a high energy consumption, the shorter VD drying time could significantly reduce energy consumption.

### 3.7. Numerical Optimization and Verification

Optimization targets minimized the total drying time, broken ratio, and SEC, as well as maximized the water-soluble polysaccharide content. The optimum operating conditions were obtained using Design Expert software (Stat-Ease). All responses were simultaneously optimized according to the goals and weights as presented in Table 5.

As shown in Table 6, the optimal conditions obtained for the given criteria were VD at 82.17 °C until the *MR* of fresh *proia cocos* cubes were reduced to 81.11%. Then, the drying was switched to IR-AID at 69.04 °C. Validation experiments were performed for *t* oaf 82 °C, *MR*_switch_ at 81%, and *T*_IR-AID_ at 69 °C. The total drying time, broken ratio, water-soluble polysaccharides content, and SEC were thus determined. Results showed the validation results were consistent with the predicted values, which proved the validity of optimization. Compared with IR-AID drying at 69 °C, the broken ratio of combined drying poria cubes decreased nearly 20%, and the drying time and SEC only increased by 58 min (0.80 MJ·kg^−1^). Compared with VD drying at 82 °C, the SEC by the two-stage drying was only one-third of the VD process. The results indicated that the two-stage VD and IR-AID process was more effective in drying poria cubes with lower energy consumption and better product qualities.

## 4. Conclusions

Fresh poria cubes were dried using the HAD, AID, IR-AID, and VD methods. To improve the drying efficiency and product qualities of poria cubes, as well as to reduce the energy consumption during the drying process, a two-stage drying was developed. It adopted advantages of VD and IR-AID. The operating conditions of the two-stage drying process were optimized as *T*_VD_ at 82 °C, *MR*_switch_ at 81%, and *T*_IR-AID_ at 69 °C for the minimized drying time (255 min), broken ratio (24.88%), and SEC (2.04 MJ·kg^−1^), as well as the maximized water-soluble polysaccharide content (3.32 mg·g^−1^). The findings from this study indicate that the new two-stage VD and IR-AID is a promising technology for improving the drying efficiency and product quality, as well as for reducing the energy consumption of poria cubes during drying. The heating source and drying method usually affect physicochemical reactions and, ultimately, the nutritional value and sensory attributes of agricultural products. The new drying technologies, which used more than one drying method, was capable of producing products with the desired moisture content and drying quality in its final products.

## Figures and Tables

**Table 1:** Coded and actual values of independent variables.

Drying Parameters		Coded Level of Variables		
		−1	0	+1
*T*_VD_ (°C)	X_1_	65	75	85
*MR*_switch_ (%)	X_2_	70	80	90
*T*_IR-AID_ (°C)	X_3_	65	75	85

**Table 2:** Drying kinetics parameters of the Dincer model and Fick’s second law.

Drying Methods	Drying Constant		*D_eff_* (m^2^·s^−1^)	$D_{eff}^{*}\;(m^{2}\cdot s^{-1})$	*Bi*	*k* (m·s^−1^)	*Adj-R* ^2^	*χ* ^2^	*RMSE*
	*G*	*S* × 10^−4^ (s^−1^)							
HAD	1.089	1.036	8.148 × 10^−9^	7.531 × 10^−9^	0.5626	6.548 × 10^−7^	0.996	1.92 × 10^−4^	0.014
AID	1.023	1.688	5.768 × 10^−8^	1.310 × 10^−8^	0.1057	8.701 × 10^−7^	0.998	1.32 × 10^−4^	0.011
IR-AID	1.022	2.197	7.901 × 10^−8^	3.767 × 10^−8^	0.1030	1.162 × 10^−6^	0.997	0.44 × 10^−4^	0.006
VD	1.087	1.044	8.401 × 10^−9^	8.335 × 10^−9^	0.5320	6.385 × 10^−7^	0.995	2.83 × 10^−4^	0.017

Note: $D_{eff}^{*}$ is the effective moisture diffusivity calculated by Fick’s second law.

**Table 3:** Box–Behnken experimental design with natural and coded drying conditions and values of response variables.

No.	Drying Conditions			Response			
	*T*_VD_ (°C)	*MR*_switch_ (%)	*T*_IR-AID_ (°C)	Total Drying Time (min)	Broken Ratio (%)	Water-Soluble Polysaccharide Content (mg·g^−1^)	SEC (MJ·kg^−1^)
1	85	90	75	187	43.50	6.77	1.88
2	75	70	85	287	5.23	10.69	2.97
3	75	80	75	245	22.10	8.10	2.25
4	75	90	85	172	44.20	4.46	1.24
5	65	70	75	325	4.35	14.54	4.04
6	85	70	75	288	3.79	10.40	2.89
7	75	90	65	228	43.79	5.83	1.62
8	75	80	75	245	21.10	8.17	2.27
9	75	80	75	248	21.70	8.10	2.25
10	75	70	65	343	4.17	12.24	3.40
11	85	80	85	212	20.90	5.36	1.49
12	65	80	85	249	21.21	8.32	2.31
13	85	80	65	256	20.52	6.70	1.86
14	75	80	75	242	21.06	8.93	2.48
15	65	90	75	213	44.10	7.31	2.03
16	65	80	65	297	20.95	9.50	2.64
17	75	80	75	251	21.37	7.70	2.14

**Table 4:** Analysis of variance (ANOVA) of the polynomial regression models for total drying time, broken ratio, water-soluble polysaccharide content, SEC.

Source	Total Drying Time (min)			Broken Ratio (%)			Water-Soluble Polysaccharide Content (mg·g^−1^)			SEC (MJ·kg^−1^)		
	DF *	Sum of Squares	*p*-Value	DF *	Sum of Squares	*p*-Value	DF *	Sum of Squares	*p*-Value	DF *	Sum of Squares	*p*-Value
*Model*	9	32,556.01	<0.0001	9	3160.99	<0.0001	9	9.71	<0.0001	9	100.69	<0.001
$x_{1}$	1	2485.13	<0.0001	1	0.45	0.1137	1	2.450 × 10^−3^	0.6237	1	13.62	<0.0001
$x_{2}$	1	24,531.12	<0.0001	1	3122.48	<0.0001	1	0.022	0.1676	1	69.08	<0.0001
$x_{3}$	1	5202.00	<0.0001	1	0.56	0.0848	1	7.33	<0.0001	1	3.69	<0.0001
$x_{1}\times x_{2}$	1	30.25	0.2892	1	4.000 × 10^−4^	0.9586	1	0.12	0.0097	1	3.24	0.0033
$x_{1}\times x_{3}$	1	4.00	0.6892	1	3.600 × 10^−3^	0.8763	1	0.67	<0.0001	1	5.184 × 10^−3^	0.0046
$x_{2}\times x_{3}$	1	0.000	1.0000	1	0.11	0.4110	1	0.18	0.0029	1	8.100 × 10^−3^	0.8745
$x_{1}^{2}$	1	9.79	0.5350	1	1.02	0.0300	1	0.30	0.0008	1	0.54	0.8435
$x_{2}^{2}$	1	128.53	0.0501	1	36.91	<0.0001	1	0.99	<0.0001	1	6.02	0.1374
$x_{3}^{2}$	1	140.42	0.0428	1	0.026	0.6750	1	0.17	0.0038	1	5.00	0.0008
Residual	7	161.05	-	7	0.97		7	0.065	-	7	1.35	-
*Lack of Fit*	3	114.25	0.1420	3	0.20	0.7903	3	0.034	0.3576	3	0.55	0.5047
*Pure Error*	4	46.80	-	4	0.76	-	4	0.031	-	4	0.80	-
*Total*	16	32,717.06	-	16	3161.96	-	16	9.78	-	16	102.04	-
*Adj- R^2^*	-	0.9887	-	-	0.9993	-	-	0.9848	-	-	0.9697	-
*Pre- R^2^*	-	0.9419	-	-	0.9986	-	-	0.9398	-	-	0.9009	-
*Adeq Precision*	-	43.968	-	-	141.174	-	-	43.991	-	-	30.168	-
*C.V.%*	-	1.90	-	-	1.65	-	-	3.20	-	-	5.22	-
*PRESS*	-	1901.12	-	-	4.44	-	-	0.59	-	-	10.11	-

Note: * DF represented degree of freedom; $x_{1}$ represented *T*_VD_, °C; $x_{2}$ represents *MR*_switch_, %; and $x_{3}$ represented *T*_IR-AID_, °C.

**Table 5:** Simultaneously optimized combined drying conditions with target and weight of investigated responses.

Response	Optimized Direction	Lower Limit	Upper Limit	Weight
Total Drying time (min)	Minimize	100	400	0.25
Broken ratio (%)	Minimize	2.0	50.0	0.25
Water-soluble polysaccharide content (mg·g^−1^)	Maximize	1.0	5.0	0.25
SEC (MJ·kg^−1^)	Minimize	1.0	5.0	0.25

**Table 6:** Prediction and validation results of response variables at the optimum condition.

Results	Operating Conditions			Response Variables			
	*T*_VD_ (°C)	*MR*_switch_ (%)	*T*_IR-AID_ (°C)	Total Drying Time (min)	Broken Ratio (%)	Water-Soluble Polysaccharide Content (mg·g^−1^)	SEC (MJ·kg^−1^)
Prediction	82.17	81.11	69.04	245.29	23.07	3.24	2.01
Validation	82	81	69	255	24.88	3.32	2.04
IR-AID	——	100	69	197	53.34	3.34	1.24
VD	82	9	——	322	3.75	4.39	6.07

## Data Availability

All the results showed in the manuscript could be requested to the corresponding author who would provide them.
